# Optimal decisions on price and inventory for a newsboy-type retailer with identifiable information and discount promotion

**DOI:** 10.1371/journal.pone.0288874

**Published:** 2023-07-21

**Authors:** Guanqun Ni

**Affiliations:** Business School, Shandong University of Technology, Zibo, Shandong, China; Chongqing University, CHINA

## Abstract

Consider a retailer selling a seasonal item, new items are stocked at the beginning of sale season and no inventory replenishment is permitted. Assuming the initial price is exogenous and the information about demand becomes more accurate as the sales season progresses, the retailer is allowed to make an in-season price adjustment after conducting a review. After the review time, if the price is adjusted to be lower than the initial price, demand increases more quickly with price decreasing which reflects the promotional effect of discount. Given the initial inventory, an optimal price adjusting model is proposed to maximize the retailer’s revenue. Taking decisions on inventory into account, the proposed model is extended to maximize the retailer’s profit rather than revenue. Numerical examples are also illustrated to test the proposed model. The results show that the optimal in-season price mainly depends on the proportion of the remaining demand, the price sensitivity, and the effect of sales promotion. An important managerial implication is that the retailer should gather the demand information about the price and raise the in-season price as soon as possible to gain more revenue when the price elasticity is small enough. Otherwise, when the price elasticity is larger, the retailer should maintain or decrease the price to gain more revenue.

## Introduction

To maximize revenue, the retailer who stocks a seasonal item for sale usually adjusts price during the sale season [1]. Examples include apparel, fashion goods, hotel rooms, airline seats, car rentals, and personal computers. There are various reasons explaining why the retailer makes price adjustment regularly, and it is incontestable that the retailer gathers more accurate information about demand as the sale season progresses, which is a process of *demand identification*. For example, Fisher et al. reported an example in the apparel industry where highly accurate demand forecasts were made after a certain *review time* [2]. With the explosive growth of global information, the data-driven analytics is developing rapidly, which can give business leaders powerful support to make decision [3]. With the application of big data and artificial intelligence, it is more convenient and possible for e-retailers to forecast and update the future demand information under an E-commerce environment [4]. In fact, the accurate demand forecasts contain two kinds of information at least: (1) target markets and their scales, and (2) demand function about price. The first kind of information describes how many potential consumers will patronize the item when the retailer keeps the same price level. While, the second kind of information depicts the demand-price curve for the whole market. Thus, there is an opportunity for the retailer to adjust price after the review time to maximize her revenue or profit.

In practice, the demand for the seasonal goods usually decreases smoothly if the new price is higher than the initial price. However, if the retailer adjusts the price to be lower than the initial price, then the demand possibly increases more drastically which reflects the promotional effect of discount, i.e., *discount promotion*. Specifically, a discount could be an important incentive for a customer and a higher discount provokes greater customer willingness to make a decision on payment. When the price is above a certain threshold, the demand varies with price smoothly, and the increasing demand due to discount only belong to the initial potential demand. When the price is lower than the threshold, discount will play a promotion role, and the increasing demand due to discount include not only the customers belonging to the initial potential demand but also the customers outside the initial potential demand [5], which is equivalent to playing the role of advertisement [6]. Regarding the second part of customers as *extra demand*, the present paper adopts a polygonal demand-price curve depicting the promotional effect of discount.

In order to illustrate the background of this study, consider a retailer who sells a seasonal item with stochastic demand. As the sales season progresses, the demand information becomes more and more accurate. Since the ordering lead time is usually longer than the sales season, like under an international supply chain environment [7], we assume that the retailer places orders before the start of the sales season and in-season replenishments are impossible. The decision-making of how much to order is based on demand estimates using pre-season historical data. Such estimates might lead to a high variance. However, as the sales season profresses, the accumulation of information and revised demand estimates might become more certain. Under the classical newsboy model, the selling price remains constant throughout the season, which prevents the retailer from increasing revenue by adjusting the selling price according to improved estimates of demand [8]. This paper extends the classical newsboy model by allowing the retailer to make an in-season price adjustment after observing partially realized demand. In practice, if the remaining stocks is more (less) than the remaining demand, the retailer can lower (raise) the price. Considering price adjustment and discount promotion, the proposed model contributes to the newsboy literature by addressing the following questions. How should the in-season price be adjusted given the initial inventory? And how much inventory should the retailer have ordered at the beginning of the sale season given the option of adjusting price later?

Considering the promotional effect of discount on extra demand, an optimal in-season price adjusting model is firstly proposed to maximize the retailer’s revenue given any initial inventory. And then, the proposed model is extended to make optimal decision on the initial inventory maximizing the retailer’s profit rather than revenue. Numerical examples are also illustrated to test the proposed model. The results show that the optimal in-season price mainly depends on the proportion of the remaining demand, the price sensitivity, and the effect of sales promotion. An important managerial implication is that the retailer should gather the demand information about the price and raise the in-season price as soon as possible to gain more revenue when the price elasticity is small enough. Otherwise, when the price elasticity is larger, the retailer should maintain or decrease the price to gain more revenue.

### Literature review

The present study has points of contact with the literature on classical newsboy model, update of forecast information, and multiperiod newsboy model. Though extant research has some limitations, the existing literature motivates the research questions. However, this paper makes important contributions to the relevant in this field.

#### Classical newsboy model

This work is closely related to the extensive literature on the newsboy problem, especially the inventory problem with a joint decision on ordering and pricing [9]. The classical newsboy problem is to find the order quantity of a product that maximizes the expected profit under a probabilistic demand [10], which can also be used in managing capacity and evaluating advantage booking of orders in some industries such as mobile phones, notebook PCs, airlines, and hotels [11]. Whitin is the first to formulate the newsboy model with price effects [12]. In previous literature, demand or market parameters are often taken to be exogenous. A literature review in [13] depicts a joint decision on ordering and pricing under stochastic additive, multiple, and united demand. Bitran and Mondschein design an optimal pricing strategy that utilizes permanent markdowns to increase demand during the selling season [14]. They show that major benefits can be derived by complementing a replenishment strategy with the dynamic adjustment of a commodity’s price as a function of its prevailing inventory and the length of its remaining sales season. Shi and Zhang consider the joint acquisition and pricing problem where the retailer sells multiple products with uncertain demands and the suppliers provide all unit quantity discounts [15]. Chen and Ho propose an analysis method for the newsboy inventory problem with fuzzy demands and incremental quantity discounts [16]. Sen and Zhang consider a model with multiple demand classes, each with a different selling price [17]. The problem is to determine how much to order and how much to allocate to each of the demand classes. The selling prices are fixed and cannot be adjusted later. Both [18, 19] permit selling price adjustments; however, the adjustments depend only on the demand rates predicted at the beginning. Realized demand information plays no role in predicting the demand for the remainder of the season. The model proposed in this paper assumes the initial inventory is a decision variable, price adjustments are allowed at a review time, and the demand after the review is a deterministic function of the adjusted price and the demand realized before the review.

#### Update of forecast information

Considering the update of forecast information, Yan and Wang assume that the seller can predict the demand for the remaining sales period based on the demand that has occurred [20]. They design an optimal in-season replenishment policy with financial constraints. Zheng et al. divide the sales period into two stages and assume that the demand information of the later stage can be accurately predicted based on the realized demand of the previous stage [21]. Considering the relationship between replenishment costs and benefits, the decision-making basis of whether the seller should replenishment in the later stage is given. Zheng et al. further assume that the demand information would be more accurate as the selling period approached and present an optimal order time [22]. Different from these studies, the present paper assumes that the future demand is not only related to the realized demand, but also affected by the promotion of future price discount.

#### Multiperiod newsboy model

Petruzzi and Dada characterize optimal ordering and pricing policies for a multiperiod, newsboy model in which demand is modeled as a constant-elastic function of price [23]. Their focus, however, is on learning the demand distribution when lost sales are not observable. Their model differs from the model proposed in the present paper because it applies to scenarios in which inventory can be replenished each period. Monahan et al. consider a multiperiod model where prices are selected for each period, but stocks are ordered for the first period only [24]. They approach this problem in a novel way by formulating a dynamic optimization model in which the demand function is isoelastic and the demands are independent. They find a strong parallel between the dynamic pricing problem and dynamic inventory models. Chew et al. jointly determine the price and the inventory allocation for a perishable product with a predetermined lifetime [25]. To maximize the expected revenue, they develop a discrete time dynamic programming model to obtain the optimal prices and the optimal inventory allocations for the product with a two period lifetime. Based on two period model, Taudes and Rudloff propose the use of an integrated pricing and inventory control model with linear demand, in which demand depends on the difference between a price-history-based reference price and the current price [26]. Considering price-dependent demand, Pal et al. study a multi-item deterministic EOQ inventory model for a vendor [27]. In their model, the authors assume that if total revenue of the vendor at any time is more than the level of price breaks, then vendor offers a percentage of discount on price to the customers.

One could view the model proposed in the present paper as a two period model, making it similar to the previous models. Nevertheless, the demand distributions for the two periods of the proposed model are not independent. On the other hand, the proposed model applies to situations where one can predict exactly an entire season’s demand after observing demands in the early part of the season. Chung et al. consider such situations and extend the classical newsboy model by allowing the retailer to make an in-season price adjustment after conducting a review; however, they assume that the price adjustment cannot influence the original market scale [28]. More realistically, the extra demand caused by the lower price could come from individuals outside the original group of potential customers. In practice, businesses always adjust the price to meet both groups of customers as the selling season progresses and often adopt some marketing promotions to attract extra demand which normally beyond the original potential demand [29–31]. As a result, the original demand function on price should be changing and becomes more complicated in the theoretical model.

The rest of this paper is organized as follows. Section 2 states the basic problem and defines notations and assumptions. There are two decision vatiables, the order quantity and the in-season price. In section 3, given inventory the solution of optimal price adjustment is derived. Section 4 deals with the problem of optimizing inventory and analyzes some numerical examples. Finally, conclusions are discussed in section 5. Some technical proofs are relegated to S1 Appendix.

## Problem formulation and notations

The problem considered in this paper can be described as follows. One retailer of seasonal goods stocks *Q* unit item at the beginning of the sale season and no inventory replenishment is permitted. The sale season starts at time 0 and ends at time *T*. To simplify the analysis, assume items unsold by time *T* have zero salvage value. At time 0, the retailer doesn’t know the demand information precisely and thus goods are sold at an exogenous competitive unit price *p*_0_ > 0. As the sale season progresses, the demand information becomes more accurate and enough additional information about demand is available at a certain review time *t*_*r*_ ∈ [0, *T*]. At time *t*_*r*_, the retailer reviews the demand during [0, *t*_*r*_], denoted by *x*_0_, and can predict demand information fairly accurately. The accurate demand information includes both demand scale and demand curve. On one hand, if the price maintains *p*_0_, then the demand scale of potential consumers has a natural relationship with the realized demand *x*_0_. In this paper, a linear function is employed to depict this relationship simply. On the other hand, if the price is adjusted, markup or markdown, the demand scale will change accordingly, which is depicted by a segmented linear demand-price curve in our model. This kind of segmented demand function implies that the influence on demand scale is distinguished at different price level. When the adjusted price is lower than the initial price, demand increases more quickly with price decreasing which reflects the promotional effect of discount. In other word, the demand caused by the new lower price come from not only the original group of potential customers, but also the individuals outside the original group of potential customers (i.e. extra demand). Based on these new information, the retailer can change the price during (*t*_*r*_, *T*] from *p*_0_ to *p*_1_ to improve revenue. Further, the retailer can optimize the initial inventory to maximize profit rather than revenue given the option of price adjustment. This paper deals with these two optimization in the following sections. The review time *t*_*r*_ depends on the characteristics of item. Both *p*_0_ and *t*_*r*_ are fixed non-decision variables in the present model. All of the main symbols that are used in the newsboy model are listed in Table 1.

**Table 1:** The symbols of the newsboy model.

Symbol	Description
*Q*	the initial inventory
*T*	the sale season
*t*_*r*_	the review time
*p*_0_	the (initial) price in [0, *t*_*r*_]
*p*_1_	the (adjusted) in-season price
*x*_0_	the realized demand in [0, *t*_*r*_]
*x*_1_	the original demand in (*t*_*r*_, *T*]
*x*	the original demand in [0, *T*]
*ω*	the extra demand in (*t*_*r*_, *T*] when *p*_1_ < *p*_0_
*D*	the total demand in [0, *T*]
λ	the demand multiplier
*α*	the absolute value of the slope of the original demand curve
*β*	the absolute value of the slope of the extra demand curve
*θ*	the price parameter

Our model imposes three assumptions on demand. First,

$$\begin{array}{c} x_{1}=\lambda x_{0}\;when\;p_{1}=p_{0}, \end{array}$$ (1)

where the demand multiplier λ is a known positive constant which reflects the natural relationship between *x*_1_ and *x*_0_. Second, *x*_1_ is a liner function of *p*_1_. Specifically,

$$\begin{array}{c} x_{1}=\lambda x_{0}-\alpha \left(x_{0}\right)\left(p_{1}-p_{0}\right), \end{array}$$ (2)

where the absolute value of original demand slope, i.e. *α*(*x*_0_), is a known non-negative constant once *x*_0_ is known. Assume *α*(*x*_0_) > 0 when *x*_0_ > 0. Third, *ω* is also a liner function of *p*_1_ when *p*_1_ < *p*_0_. Referring to [29], the extra demand *ω* is also defined in terms of the proportion of demand *x*_0_. However, the absolute value of extra demand slope, i.e. *β*(*x*_0_), should be less than *α*(*x*_0_) intuitively, since the extra demand could not be more than the demand of the original potential population. Specifically *ω* = *β*(*x*_0_)(*p*_0_ − *p*_1_), when *p*_1_ < *p*_0_, or else *ω* = 0. Thus, the extra demand can be described as

$$\begin{array}{c} \omega =\beta \left(x_{0}\right)\;\text{max}\left\{\left(p_{0}-p_{1}\right),0\right\}, \end{array}$$ (3)

where *β*(*x*_0_) is a known non-negative constant once *x*_0_ is known. Assume *β*(*x*_0_) > 0 when *x*_0_ > 0.

Two types of *α*-functions are of special interest, constant functions with *α*(*x*_0_) = *α* for all *x*_0_, and proportional functions with *α*(*x*_0_) = *αx*_0_ for all *x*_0_, where *α* is a known positive constant. The latter type seems more applicable, since it accounts for the scale of the demand [28]. The present model also employs proportional functions for *α*(*x*_0_) and *β*(*x*_0_), that is, *α*(*x*_0_) = *αx*_0_ and *β*(*x*_0_) = *βx*_0_.

The decision on *p*_1_ is made after both *p*_0_ and *x*_0_ are known. One can represent the price and demand changes in terms of a price parameter *θ* as follows.

$$\begin{array}{c} p_{1}=p_{1}\left(\theta \right)=\left(1+\theta \right)p_{0}, \end{array}$$ (4)

$$\begin{array}{c} x_{1}=x_{1}\left(\theta \right)=\lambda x_{0}-\alpha x_{0}\theta p_{0}, \end{array}$$ (5)

$$\begin{array}{c} x\left(\theta \right)=x_{0}+x_{1}\left(\theta \right)=\left(1+\lambda \right)x_{0}-\alpha x_{0}\theta p_{0}, \end{array}$$ (6)

$$\begin{array}{c} \omega \left(\theta \right)=\beta \left(x_{0}\right)\;\text{max}\left\{\left(p_{0}-p_{1}\right),0\right\}=\beta x_{0}p_{0}\;\text{max}\left\{-\theta ,0\right\}, \end{array}$$ (7)

$$\begin{array}{c} D\left(\theta \right)=x\left(\theta \right)+\omega \left(\theta \right)=\left(1+\lambda \right)x_{0}-\alpha x_{0}\theta p_{0}+\beta x_{0}p_{0}\;\text{max}\left\{-\theta ,0\right\}. \end{array}$$ (8)

Specifically, the demand function can be expressed as a piecewise function of price and it may be more than two segments which doesn’t break down the generality of results. For each segment, there is a unique demand-price expression and the expression is usually different among price intervals by means of different price elasticity of demand. In reality, this kind of situation is common. For example, when the price is very high, only a few highest earners maybe become effective potential customers and the demand function about price may be like Eq (6) as the proposed model. When the price is not high enough, there will be a few higher earners becoming part of effective potential customers. When the price is further lower, the effective potential customers will contain more low-income groups. When the price is very low, the scale of effective potential customers will further increase. In a real world, there might be two reasons explaining this phenomenon: (1) the sensitivities of price are different for different income level of customers, and (2) different prices have different effect on sales promotion. The present model considers these two reasons and the main consideration is the latter one. Specifically, when the price is higher than some threshold, the promotional effect of discount on sale is not obvious. However when the price is lower than the threshold, the promotional effect is much obvious.

As above statement, the initial price maybe not just is the threshold price. However, it does not affect the generality of the results. In the following text, the demand caused by the promotional effect of discount is regarded as the extra demand which does not belong to the initial scale of potential demand. In this model, the parameter *β* reflects the effect of sales promotion and the demand caused by this factor are corresponding to the extra demand. The parameter *α* just reflects the relationship between demand and price in the initial scale of potential demand. In real life, one can gather the information about promotional effect of discount by marketing method, and further, find out the relationship between the extra demand and the discount precisely. Additionally, the real relationship between demand and price should usually be expressed as the joint of more than one segmented smooth demand-price curves. In the present model, the demand function of price is segmented which might be contribute to this solution.

Assuming there are enough inventory of item, i.e. *D*(*θ*) ≤ *Q*, the revenue from sales in (*t*_*r*_, *T*] equals

$$\begin{array}{c} \begin{array}{c} p_{1}\left(\theta \right)\left(x_{1}\left(\theta \right)+\omega \left(\theta \right)\right)=\{\begin{array}{c} -\alpha x_{0}p_{0}^{2}\theta^{2}+\left(\lambda x_{0}p_{0}-\alpha x_{0}p_{0}^{2}\right)\theta +\lambda x_{0}p_{0},\;if\;\theta >0\\ -\left(\alpha +\beta \right)x_{0}p_{0}^{2}\theta^{2}+\left[\lambda x_{0}p_{0}-\left(\alpha +\beta \right)x_{0}p_{0}^{2}\right]\theta +\lambda x_{0}p_{0},\;if\;\theta \leq 0 \end{array}, \end{array} \end{array}$$ (9)

Thus, the total revenue *R*(*θ*) for [0, *T*] satisfies

$$\begin{array}{c} R\left(\theta \right)=\{\begin{array}{c} q_{1}\left(\theta \right)p_{0},\;if\;\theta >0\\ q_{2}\left(\theta \right)p_{0},\;if\;\theta \leq 0 \end{array}, \end{array}$$ (10)

where for any real *θ*

$$\begin{array}{c} q_{1}\left(\theta \right)=-\alpha x_{0}p_{0}\theta^{2}+\left(\lambda x_{0}-\alpha x_{0}p_{0}\right)\theta +\left(1+\lambda \right)x_{0}, \end{array}$$ (11)

$$\begin{array}{c} q_{2}\left(\theta \right)=-\left(\alpha +\beta \right)x_{0}p_{0}\theta^{2}+\left[\lambda x_{0}-\left(\alpha +\beta \right)x_{0}p_{0}\right]\theta +\left(1+\lambda \right)x_{0}. \end{array}$$ (12)

Based on the assumption that *α* > *β* > 0, *q*_1_(*θ*) and *q*_2_(*θ*) are concave quadratic functions and *q*_1_(0) = *q*_2_(0) = (1 + λ)*x*_0_. Hence, for *j* = 1 and 2, *q*_*j*_(⋅) is maximized at its axis of symmetry, i.e. $\theta_{j}^{\text{max}}$, where

$$\begin{array}{c} \theta_{1}^{\text{max}}=\frac{1}{2}\left(\frac{\lambda }{\alpha p_{0}}-1\right), \end{array}$$ (13)

and

$$\begin{array}{c} \theta_{2}^{\text{max}}=\frac{1}{2}\left(\frac{\lambda }{\left(\alpha +\beta \right)p_{0}}-1\right). \end{array}$$ (14)

Of course

$$\begin{array}{c} \theta_{1}^{\text{max}}\geq \theta_{2}^{\text{max}}. \end{array}$$ (15)

## Optimal price adjustment given *Q*

For *x*_0_ ≥ 0 and *Q* ≥ 0, define θ*(*x*_0_, *Q*) the value of *θ* resulting a maximum revenue given *x*_0_ and *Q*. This section characterizes θ*(*x*_0_, *Q*). Considering the problem of maximizing revenue, the situation of *x*_0_ ≥ *Q* is trivial, since there are no sales in (*t*_*r*_, *T*]. Therefore, the following analysis focuses on the situation of *x*_0_ < *Q*. First, the analysis requires the lemma below.

**Lemma 1** *Suppose α* > *β* > 0.

*(a)* $\theta_{1}^{\text{max}}<0$ *if and only if* $\frac{\lambda }{\alpha p_{0}}<1$, *and* $\theta_{2}^{\text{max}}>0$ *if and only if* $\frac{\lambda }{\left(\alpha +\beta \right)p_{0}}>1$.
*(b) Suppose* $\frac{\lambda }{\alpha p_{0}}>\frac{\lambda }{\left(\alpha +\beta \right)p_{0}}>1$. *Then* $\theta_{1}^{\text{max}}>\theta_{2}^{\text{max}}>0$. *Furthermore, R*(*θ*) *is an increasing function of θ on* $\left(-\infty ,\theta_{1}^{\text{max}}\right]$ *and a decreasing function of θ on* $\left[\theta_{1}^{\text{max}},+\infty \right)$.
*(c) Suppose* $\frac{\lambda }{\alpha p_{0}}>1>\frac{\lambda }{\left(\alpha +\beta \right)p_{0}}$. *Then* $\theta_{1}^{\text{max}}>0>\theta_{2}^{\text{max}}$. *Furthermore, R*(*θ*) *is an increasing function of θ on* $\left(-\infty ,\theta_{2}^{\text{max}}\right]$ *and* $\left[0,\theta_{1}^{\text{max}}\right]$, *and a decreasing function of θ on* $\left[\theta_{2}^{\text{max}},0\right]$ *and* $\left[\theta_{1}^{\text{max}},+\infty \right)$.
*(d) Suppose* $1>\frac{\lambda }{\alpha p_{0}}>\frac{\lambda }{\left(\alpha +\beta \right)p_{0}}$. *Then* $0>\theta_{1}^{\text{max}}>\theta_{2}^{\text{max}}$. *Furthermore, R*(*θ*) *is an increasing function of θ on* $\left(-\infty ,\theta_{2}^{\text{max}}\right]$ *and a decreasing function of θ on* $\left[\theta_{2}^{\text{max}},+\infty \right)$.

**Proof 1 (Proof of Lemma 1)** *First, part (a) is obvious directly from Eqs* (13) *and* (14). *Next, consider part (b). Part (a) and* Eq (15) *imply* $\theta_{1}^{\text{max}}>\theta_{2}^{\text{max}}>0$ *when* $\frac{\lambda }{\alpha p_{0}}>\frac{\lambda }{\left(\alpha +\beta \right)p_{0}}>1$. *Let θ* > 0. *Then R*(*θ*) = *q*_1_(*θ*)*p*_0_. *Clearly, q*_1_(*θ*) *is increasing on* $\left(-\infty ,\theta_{1}^{\text{max}}\right]$ *and decreasing on* $\left[\theta_{1}^{\text{max}},+\infty \right)$. *Since* $\theta_{1}^{\text{max}}>0$, *R*(*θ*) *is increasing on* $\left(0,\theta_{1}^{\text{max}}\right]$ *and decreasing on* $\left[\theta_{1}^{\text{max}},+\infty \right)$. *Let θ* ≤ 0. *Then R*(*θ*) = *q*_2_(*θ*)*p*_0_. *Clearly, q*_2_(*θ*) *is increasing on* $\left(-\infty ,\theta_{2}^{\text{max}}\right]$ *and decreasing on* $\left[\theta_{2}^{\text{max}},+\infty \right)$. *Since* $\theta_{2}^{\text{max}}>0$, *R*(*θ*) *is increasing on* (−∞, 0]. *This proves part (b)*.

*For part (c), part (a) and* Eq (15) *imply* $\theta_{1}^{\text{max}}>0>\theta_{2}^{\text{max}}$ *when* $\frac{\lambda }{\alpha p_{0}}>1>\frac{\lambda }{\left(\alpha +\beta \right)p_{0}}$ . *If θ* > 0, *then R*(*θ*) = *q*_1_(*θ*)*p*_0_. *Since* $\theta_{1}^{\text{max}}>0$, *R*(*θ*) *is increasing on* $\left[0,\theta_{1}^{\text{max}}\right]$ *and decreasing on* $\left[\theta_{1}^{\text{max}},+\infty \right)$. *If θ* ≤ 0, *then R*(*θ*) = *q*_2_(*θ*)*p*_0_. *Since* $\theta_{2}^{\text{max}}<0$, *R*(*θ*) *is increasing on* $\left(-\infty ,\theta_{2}^{\text{max}}\right]$ *and decreasing on* $\left[\theta_{2}^{\text{max}},0\right]$. *This proves part (c). Similar arguments prove part (d)*.

For *x*_0_ ≥ 0 and *Q* ≥ 0, define

$$\begin{array}{c} \theta_{0}=\underset{\theta }{\text{arg}}\left(D\left(\theta \right)=Q\right) \end{array}$$ (16)

For the value of *θ*_0_, one can show that

$$\begin{array}{c} IfD\left(0\right)\leq Q,\theta_{0}=\theta_{0}^{-}=\frac{\left(1+\lambda \right)x_{0}-Q}{\left(\alpha +\beta \right)x_{0}p_{0}}\leq 0, \end{array}$$ (17)

$$\begin{array}{c} IfD\left(0\right)>Q,\theta_{0}=\theta_{0}^{+}=\frac{\left(1+\lambda \right)x_{0}-Q}{\alpha x_{0}p_{0}}>0. \end{array}$$ (18)

And the following two theorems can be derived.

**Theorem 1** *Suppose* *D*(0) ≤ *Q* *and* *α* > *β* > 0.

*(a) The revenue equals R*(*θ*) *for θ* ∈ [*θ*_0_, + ∞), *and θ*_0_ ≤ 0.
*(b) If* $\frac{\lambda }{\alpha p_{0}}>\frac{\lambda }{\left(\alpha +\beta \right)p_{0}}>1$, *then* $\theta^{*}\left(x_{0},Q\right)=\theta_{1}^{\text{max}}$.
*(c) If* $\frac{\lambda }{\alpha p_{0}}>1>\frac{\lambda }{\left(\alpha +\beta \right)p_{0}}$, *then* $\theta^{*}\left(x_{0},Q\right)=\theta_{1}^{\text{max}}$ *or* $=\;\text{max}\{\theta_{2}^{\text{max}},\theta_{0}^{-}\}$ *depending on the resulting revenue*.
*(d) If* $1>\frac{\lambda }{\alpha p_{0}}>\frac{\lambda }{\left(\alpha +\beta \right)p_{0}}$, *then* $\theta^{*}\left(x_{0},Q\right)=\;\text{max}\left\{\theta_{2}^{\text{max}},\theta_{0}^{-}\right\}$.

**Proof 2 (Proof of Theorem 1)** *For part (a), D*(0) ≤ *Q and* Eq (17) *imply θ*_0_ ≤ 0. *Clearly, the value of R*(*θ*) *when θ* < *θ*_0_ *is less than that when* *θ* = *θ*_0_. *Because it is the case that D*(*θ*) > *D*(*θ*_0_) = *Q and p*_1_(*θ*) < *p*_1_(*θ*_0_) *when θ* < *θ*_0_, *the quantity of item sold out is at most equal to Q*. *Thus, the resulting revenue R*(*θ*) = *p*_0_*x*_0_ + (*Q* − *x*_0_)*p*_1_(*θ*) < *p*_0_*x*_0_ + (*Q* − *x*_0_)*p*_1_(*θ*_0_) = *R*(*θ*_0_).

*Consider part (b). Part (a) and Lemma 1 (b) imply R*(*θ*) *is an increasing function of θ on* $\left[\theta_{0},\theta_{1}^{\text{max}}\right]$ *and a decreasing function of θ on* $\left[\theta_{1}^{\text{max}},+\infty \right)$. *This proves part (b)*.

*Next for part (c), using Lemma 1 (c) the retailer can increase revenue by decreasing θ away from* 0 *until* $\theta =\theta_{0}^{-}$ *or θ reaches* $\theta_{2}^{\text{max}}$ , *whichever occurs first, or by increasing θ away from* 0 *until* $\theta =\theta_{1}^{\text{max}}$. *By comparing revenues, the retailer chooses the value of θ which results in more revenue. This proves part (c). Based on the proof of part (c), part (d) follows directly from part (d) of Lemma 1*.

**Theorem 2** *Suppose D*(0) > *Q and α* > *β* > 0.

*(a) The revenue equals R*(*θ*) *for θ* ∈ [*θ*_0_, + ∞), *and θ*_0_ > 0.
*(b) If* $\frac{\lambda }{\alpha p_{0}}>\frac{\lambda }{\left(\alpha +\beta \right)p_{0}}>1$, *then* $\theta^{*}\left(x_{0},Q\right)=\;\text{max}\left\{\theta_{1}^{\text{max}},\theta_{0}^{+}\right\}$.
*(c) If* $\frac{\lambda }{\alpha p_{0}}>1>\frac{\lambda }{\left(\alpha +\beta \right)p_{0}}$, *then* $\theta^{*}\left(x_{0},Q\right)=\;\text{max}\left\{\theta_{1}^{\text{max}},\theta_{0}^{+}\right\}$.
*(d) If* $1>\frac{\lambda }{\alpha p_{0}}>\frac{\lambda }{\left(\alpha +\beta \right)p_{0}}$, *then* $\theta^{*}\left(x_{0},Q\right)=\theta_{0}^{+}$.

**Proof 3 (Proof of Theorem 2)** *For part (a), it is similar to Theorem 1. Therefore, parts (b), (c), and (d) follow directly from parts (b), (c), and (d) of Lemma 1*.

Theorem 1 shows that if the initial ordering quantity is no less than the actual demand when the price is fixed at the initial price, then it is the case that *θ*_0_ ≤ 0 and the price should not be lower than *p*_0_(1+ *θ*_0_). Otherwise, the resulting revenue is definitely not the optimal one. For the parts (b), (c), and (d), the results depend on the values of $\frac{\lambda }{\alpha p_{0}}$ and $\frac{\lambda }{\left(\alpha +\beta \right)p_{0}}$. In particular, the numerators of these two fractions are the same and reflect the time of review *t*_*r*_. The larger the value of λ, the greater proportion of the following demand, that is, the earlier the review time *t*_*r*_. For the fraction $\frac{\lambda }{\alpha p_{0}}$, the denominator equals the product of the slope and the initial price which could be understood as the price sensitivity in the initial demand scale, that is, the larger the denominator, the more sensitive the customers. For the fraction $\frac{\lambda }{\left(\alpha +\beta \right)p_{0}}$, the denominator contains two parts, the first part *αp*_0_ has the same meaning with the fraction $\frac{\lambda }{\alpha p_{0}}$ and the second part *βp*_0_ reflects the effect of sales promotion. The larger the value of *β*, the more obvious the promotion effect.

Part (b) of Theorem 1 shows that when $\frac{\lambda }{\alpha p_{0}}>\frac{\lambda }{\left(\alpha +\beta \right)p_{0}}>1$ which is resulted by weaker promotion effect (smaller value of *β*) or greater proportion of the following demand (larger value of λ), the optimal decision should be to raise price and the optimal price is just equal to $p_{0}\left(1+\theta_{1}^{\text{max}}\right)$. More intuitively, when the discount doesn’t bring enough extra customers, the discount policy cannot increase the profit. Thus, the optimal decision should be raising the price.

Part (c) of Theorem 1 shows that when $\frac{\lambda }{\alpha p_{0}}>1>\frac{\lambda }{\left(\alpha +\beta \right)p_{0}}$ which is resulted by more forceful promotion effect (larger value of *β*) or mild proportion of the following demand (moderate value of λ), the decision maker maybe have two choices. On the one hand, considering the less scale of remaining demand, the decision maker might raise the price to $p_{0}\left(1+\theta_{1}^{\text{max}}\right)$. On the other hand, considering the forceful promotion effect of discount, the decision maker might reduce the price. Combining with part (a), the optimal price should be equal to $p_{0}\left(1+\text{max}\left\{\theta_{2}^{\text{max}},\theta_{0}^{-}\right\}\right)$ for the second choice. The price is chosen depending on the specific value of parameters for the case of part (c).

Part (d) of Theorem 1 shows that if the time of review is late enough, then the optimal decision is reducing the price to $p_{0}\left(1+\;\text{max}\left\{\theta_{2}^{\text{max}},\theta_{0}^{-}\right\}\right)$.

Theorem 2 shows that if the initial ordering quantity is less than the actual demand when the price is fixed at the initial price, then it is the case that *θ*_0_ > 0 and the price should not be lower than *p*_0_(1+ *θ*_0_), that is, the optimal decision should be raising the price. Part (b) shows that when the promotion effect is weaker or the proportion of the following demand is greater, the optimal decision should be to raise price and the optimal price is just equal to $p_{0}\left(1+\;\text{max}\left\{\theta_{1}^{\text{max}},\theta_{0}^{+}\right\}\right)$ similar to the analysis of Theorem 1.

Part (c) of Theorem 2 shows that when the promotion effect is forceful or the proportion of the following demand is mild, the optimal price should also be equal to $p_{0}\left(1+\;\text{max}\left\{\theta_{1}^{\text{max}},\theta_{0}^{+}\right\}\right)$. In short, the optimal price of parts (b) and (c) are both equal to $p_{0}\left(1+\;\text{max}\left\{\theta_{1}^{\text{max}},\theta_{0}^{+}\right\}\right)$. Under the situation of Theorem 2, if the ordering quantity is much less than the initial potential demand and then the value of $\theta_{0}^{+}$ will be bigger than the value of $\theta_{1}^{max}$ according to the expressions of (13) and (18). Therefore, the optimal price should be $p_{0}\left(1+\theta_{0}^{+}\right)$ based on Lemma 1. Otherwise, if the decision maker chooses the price equal to $p_{0}\left(1+\theta_{1}^{max}\right)$, then the ordering quantity cannot meet all the demand, that is, the optimal price should just equal $p_{0}\left(1+\theta_{0}^{+}\right)$. On the other hand, if the ordering quantity is mild less than the initial potential demand and then the value of $\theta_{0}^{+}$ will be smaller than the value of $\theta_{1}^{max}$. Therefore, the optimal price should be $p_{0}\left(1+\theta_{1}^{max}\right)$. Generally speaking, the optimal price for parts (b) and (c) should both be $p_{0}\left(1+\;\text{max}\left\{\theta_{1}^{\text{max}},\theta_{0}^{+}\right\}\right)$.

Part (d) of Theorem 2 shows that if the time of review is late enough, then the optimal price should be equal to $p_{0}\left(1+\theta_{0}^{+}\right)$. Because when $1>\frac{\lambda }{\alpha p_{0}}>\frac{\lambda }{\left(\alpha +\beta \right)p_{0}}$, based on above analysis, it must be the case that $\theta_{0}^{+}>\theta_{1}^{max}$.

The next lemma, which follows easily from (8) and (16), allows one to apply Theorems 1 and 2 and to get an expression for the optimal revemue as a function of *Q*.

**Lemma 2** *For any Q* ≥ 0 *and real θ*, *θ*_0_ ≥ *θ* *is equivalent to Q* ≤ *D*(*θ*).

There are three cases appearing in Theorems 1 and 2.

Case 1. $\frac{\lambda }{\alpha p_{0}}>\frac{\lambda }{\left(\alpha +\beta \right)p_{0}}>1$. Here $\theta_{1}^{\text{max}}>\theta_{2}^{\text{max}}>0$. This case is broken down into two subcases.

Case 1a. *D*(0) ≤ *Q*. Using Theorem 1 (b) and Lemma 2, one can show that

$$\begin{array}{c} \theta^{*}\left(x_{0},Q\right)=\theta_{1}^{\text{max}}. \end{array}$$ (19)

Case 1b. *D*(0) > *Q*. Using Theorem 2 (b) and Lemma 2, one can show that

$$\begin{array}{c} \theta^{*}\left(x_{0},Q\right)=\{\begin{array}{c} \theta_{0}^{+},\;\;x_{0}\leq Q\leq \Delta_{1}\\ \theta_{1}^{\text{max}},\;\Delta_{1}\leq Q<D\left(0\right) \end{array}, \end{array}$$ (20)

where

$$\begin{array}{c} \Delta_{1}=\left(1+\lambda \right)x_{0}+\frac{x_{0}}{2}\left(\alpha p_{0}-\lambda \right). \end{array}$$ (21)

First, because it is the case that $\frac{\lambda }{\alpha p_{0}}>1$, we have *x*_0_ < Δ_1_ < *D*(0) = (1 + λ)*x*_0_. When *Q* = Δ_1_, one can calculate that $\theta_{0}^{+}=\frac{1}{2}\left(\frac{\lambda }{\alpha p_{0}}-1\right)=\theta_{1}^{\text{max}}$ from Eqs (13) and (18). Using Lemma 2, one can show that $\theta_{0}^{+}\geq \theta_{1}^{\text{max}}$ when *x*_0_ ≤ *Q* ≤ Δ_1_ and $\theta_{0}^{+}\leq \theta_{1}^{\text{max}}$ when Δ_1_ ≤ *Q* < *D*(0). This proves Case 1b.

Using Eqs (19) and (20), one can easily establish that the following hold for Case 1, where θ*(⋅) is the optimal price adjutment and *g*(⋅) is the corresponding maximum revenue function.

$$\begin{array}{c} \theta^{*}\left(x_{0},Q\right)=\{\begin{array}{c} \theta_{0}^{+},\;\;x_{0}\leq Q\leq \Delta_{1}\\ \theta_{1}^{\text{max}},\;\Delta_{1}\leq Q \end{array}, \end{array}$$ (22)

$$\begin{array}{c} g\left(x_{0},Q\right)=\{\begin{array}{c} q_{1}\left(\theta_{0}^{+}\right)p_{0},\;\;x_{0}\leq Q\leq \Delta_{1}\\ q_{1}\left(\theta_{1}^{\text{max}}\right)p_{0},\;\Delta_{1}\leq Q \end{array}. \end{array}$$ (23)

Case 2. $\frac{\lambda }{\alpha p_{0}}>1>\frac{\lambda }{\left(\alpha +\beta \right)p_{0}}$. Here $\theta_{1}^{\text{max}}>0>\theta_{2}^{\text{max}}$. This case is also broken down into two subcases.

Case 2a. *D*(0) ≤ *Q*. In this case, the retailer may increase or decrease the selling price to gain maximal revenue based on Theorem 1 (c). Please see S1 Appendix for the proof of the following result.

If $\alpha \left(\alpha +\beta \right)p_{0}^{2}\geq \lambda^{2}$, it is the case that

$$\begin{array}{c} \theta^{*}\left(x_{0},Q\right)=\{\begin{array}{c} \theta_{1}^{\text{max}},\;D\left(0\right)\leq Q\leq \Delta_{3}\\ \theta_{0}^{-},\;\Delta_{3}\leq Q\leq \Delta_{2}\\ \theta_{2}^{\text{max}},\;\Delta_{2}\leq Q \end{array}, \end{array}$$ (24)

$$\begin{array}{c} g\left(x_{0},Q\right)=\{\begin{array}{c} q_{1}\left(\theta_{1}^{\text{max}}\right)p_{0},\;D\left(0\right)\leq Q\leq \Delta_{3}\\ q_{2}\left(\theta_{0}^{-}\right)p_{0},\;\Delta_{3}\leq Q\leq \Delta_{2}\\ q_{2}\left(\theta_{2}^{\text{max}}\right)p_{0},\;\Delta_{2}\leq Q \end{array}, \end{array}$$ (25)

$$\begin{array}{c} \Delta_{2}=\left(1+\lambda \right)x_{0}+\frac{x_{0}}{2}\left[\left(\alpha +\beta \right)p_{0}-\lambda \right], \end{array}$$ (26)

$$\begin{array}{c} \Delta_{3}=\frac{\left(\alpha +\beta \right)x_{0}}{2}\left[p_{0}+\frac{\lambda +2}{\alpha +\beta }-\sqrt{\frac{\beta }{\alpha +\beta }\left(p_{0}^{2}-\frac{\lambda^{2}}{\alpha (\alpha +\beta )}\right)}\right]. \end{array}$$ (27)

If $\alpha \left(\alpha +\beta \right)p_{0}^{2}<\lambda^{2}$, it is the case that

$$\begin{array}{c} \theta^{*}\left(x_{0},Q\right)=\theta_{1}^{\text{max}}, \end{array}$$ (28)

$$\begin{array}{c} g\left(x_{0},Q\right)=q_{1}\left(\theta_{1}^{\text{max}}\right)p_{0}. \end{array}$$ (29)

Case 2b. *D*(0) > *Q*. As Case 1b, using Theorem 2 (c) and Lemma 2 one can show that

$$\begin{array}{c} \theta^{*}\left(x_{0},Q\right)=\{\begin{array}{c} \theta_{0}^{+},\;\;x_{0}\leq Q\leq \Delta_{1}\\ \theta_{1}^{\text{max}},\;\Delta_{1}\leq Q<D\left(0\right) \end{array}, \end{array}$$ (30)

Based on above analysis, the following hold for Case 2.

If $\alpha \left(\alpha +\beta \right)p_{0}^{2}\geq \lambda^{2}$, it is the case that

$$\begin{array}{c} \theta^{*}\left(x_{0},Q\right)=\{\begin{array}{c} \theta_{0}^{+},\;x_{0}\leq Q\leq \Delta_{1}\\ \theta_{1}^{\text{max}},\;\Delta_{1}\leq Q\leq \Delta_{3}\\ \theta_{0}^{-},\;\Delta_{3}\leq Q\leq \Delta_{2}\\ \theta_{2}^{\text{max}},\;\Delta_{2}\leq Q \end{array}, \end{array}$$ (31)

$$\begin{array}{c} g\left(x_{0},Q\right)=\{\begin{array}{c} q_{1}\left(\theta_{0}^{+}\right)p_{0},\;x_{0}\leq Q\leq \Delta_{1}\\ q_{1}\left(\theta_{1}^{\text{max}}\right)p_{0},\;\Delta_{1}\leq Q\leq \Delta_{3}\\ q_{2}\left(\theta_{0}^{-}\right)p_{0},\;\Delta_{3}\leq Q\leq \Delta_{2}\\ q_{2}\left(\theta_{2}^{\text{max}}\right)p_{0},\;\Delta_{2}\leq Q \end{array}. \end{array}$$ (32)

If $\alpha \left(\alpha +\beta \right)p_{0}^{2}<\lambda^{2}$, it is the case that

$$\begin{array}{c} \theta^{*}\left(x_{0},Q\right)=\{\begin{array}{c} \theta_{0}^{+},\;x_{0}\leq Q\leq \Delta_{1}\\ \theta_{1}^{\text{max}},\;\Delta_{1}\leq Q \end{array}, \end{array}$$ (33)

$$\begin{array}{c} g\left(x_{0},Q\right)=\{\begin{array}{c} q_{1}\left(\theta_{0}^{+}\right)p_{0},\;x_{0}\leq Q\leq \Delta_{1}\\ q_{1}\left(\theta_{1}^{\text{max}}\right)p_{0},\;\Delta_{1}\leq Q \end{array}. \end{array}$$ (34)

Case 3. $1>\frac{\lambda }{\alpha p_{0}}>\frac{\lambda }{\left(\alpha +\beta \right)p_{0}}$. Here $0>\theta_{1}^{\text{max}}>\theta_{2}^{\text{max}}$. If *D*(0) ≤ *Q*, then Theorem 1 (d) implies that $\theta^{*}\left(x_{0},Q\right)=\;\text{max}\left\{\theta_{2}^{\text{max}},\theta_{0}^{-}\right\}$. As Case 2a, one can show that

$$\begin{array}{c} \theta^{*}\left(x_{0},Q\right)=\{\begin{array}{c} \theta_{0}^{-},\;D(0)\leq Q\leq \Delta_{2}\\ \theta_{2}^{\text{max}},\;\Delta_{2}\leq Q \end{array}, \end{array}$$ (35)

If *D*(0) > *Q*, then Theorem 2 (d) implies that

$$\begin{array}{c} \theta^{*}\left(x_{0},Q\right)=\theta_{0}^{+}. \end{array}$$ (36)

Thus, the following hold for Case 3.

$$\begin{array}{c} \theta^{*}\left(x_{0},Q\right)=\{\begin{array}{c} \theta_{0}^{+},\;x_{0}\leq Q\leq D\left(0\right)\\ \theta_{0}^{-},\;D\left(0\right)\leq Q\leq \Delta_{2}\\ \theta_{2}^{\text{max}},\;\Delta_{2}\leq Q \end{array}, \end{array}$$ (37)

$$\begin{array}{c} g\left(x_{0},Q\right)=\{\begin{array}{c} q_{1}\left(\theta_{0}^{+}\right)p_{0},\;x_{0}\leq Q\leq D\left(0\right)\\ q_{2}\left(\theta_{0}^{-}\right)p_{0},\;D\left(0\right)\leq Q\leq \Delta_{2}\\ q_{2}\left(\theta_{2}^{\text{max}}\right)p_{0},\;\Delta_{2}\leq Q \end{array}. \end{array}$$ (38)

## Inventory decision with stochastic *x*_0_

Given any *Q* ≥ *x*_0_ ≥ 0, we have resolve the optimal price adjustment in Section. Of course, one can calculate the optimal initial inventory *Q* maximizing the revenue given *x*_0_. However, the quantity sold before the review time *t*_*r*_, i.e. the value of *x*_0_, is usually uncertain or stochastic in practice, although the relationship between *x*_0_ and *x*_1_, i.e. the value of λ, is known in advance. For example, a retailer selling summer apparel can fairly forecast the relation of sales volumes between the first month and the following months, no matter how much is the volume of the first month, based on demand history. Therefore, it is an important task for the retailer to optimize the price adjustment *θ*, together with the initial inventory *Q*, under the stochastic assumption of *x*_0_. Considering the purchase cost, the present joint problem to maximize the profit rather than the revenue is discussed in this section.

Define *c* the unit purchase cost and let *f*(*x*) and *F*(*x*) be the density function and cumulative distribution function of *x*_0_ respectively, then the profit function can be show as

$$\begin{array}{c} H\left(Q\right)=-cQ+p_{0}Q\left(1-F\left(Q\right)\right)+\int_{0}^{Q}g(x,Q)f(x)dx. \end{array}$$ (39)

An optimal inventory quantity Q* solves the following optimization problem,

$$\begin{array}{c} \begin{array}{c} Maximize\;H(Q)\\ Subject\;to:\;Q\geq 0 \end{array} \end{array}$$ (40)

Let $h\left(Q\right)=\int_{0}^{Q}g(x,Q)\;f(x)dx$, then each case in Section 3 has its own expression of *h*(*Q*) as follows.

For Case 1, $\frac{\lambda }{\alpha p_{0}}>\frac{\lambda }{\left(\alpha +\beta \right)p_{0}}>1$,

$$\begin{array}{c} h\left(Q\right)=\int_{0}^{\eta_{1}\left(Q\right)}q_{1}\left(\theta_{1}^{max}\right)p_{0}\;f\left(x\right)dx+\int_{\eta_{1}\left(Q\right)}^{Q}q_{1}\left(\theta_{0}^{+}\right)p_{0}\;f\left(x\right)dx, \end{array}$$ (41)

where $\eta_{1}\left(Q\right)=\frac{Q}{\left(1+\lambda \right)+\frac{1}{2}\left(\alpha p_{0}-\lambda \right)}=\frac{Q}{\eta_{1}}$.

For Case 2, $\frac{\lambda }{\alpha p_{0}}>1>\frac{\lambda }{\left(\alpha +\beta \right)p_{0}}$, if $\alpha \left(\alpha +\beta \right)p_{0}^{2}\geq \lambda^{2}$, then

$$\begin{array}{c} \begin{array}{c} h\left(Q\right)=\int_{0}^{\eta_{2}\left(Q\right)}q_{2}\left(\theta_{2}^{max}\right)p_{0}\;f\left(x\right)dx+\int_{\eta_{2}\left(Q\right)}^{\eta_{3}\left(Q\right)}q_{2}\left(\theta_{0}^{-}\right)p_{0}\;f\left(x\right)dx\\ \;\;\;\;+\int_{\eta_{3}\left(Q\right)}^{\eta_{1}\left(Q\right)}q_{1}\left(\theta_{1}^{max}\right)p_{0}\;f\left(x\right)dx+\int_{\eta_{1}\left(Q\right)}^{Q}q_{1}\left(\theta_{0}^{+}\right)p_{0}\;f\left(x\right)dx, \end{array} \end{array}$$ (42)

where $\eta_{2}\left(Q\right)=\frac{Q}{\left(1+\lambda \right)+\frac{1}{2}\left[\left(\alpha +\beta \right)p_{0}-\lambda \right]}=\frac{Q}{\eta_{2}}$ and $\eta_{3}\left(Q\right)=\frac{Q}{\frac{\alpha +\beta }{2}\left[p_{0}+\frac{2+\lambda }{\alpha +\beta }-\sqrt{\frac{\beta }{\alpha +\beta }\left(p_{0}^{2}-\frac{\lambda^{2}}{\alpha (\alpha +\beta )}\right)}\right]}=\frac{Q}{\eta_{3}}$.

For Case 2, $\frac{\lambda }{\alpha p_{0}}>1>\frac{\lambda }{\left(\alpha +\beta \right)p_{0}}$, if $\alpha \left(\alpha +\beta \right)p_{0}^{2}<\lambda^{2}$, then

$$\begin{array}{c} h\left(Q\right)=\int_{0}^{\eta_{1}\left(Q\right)}q_{1}\left(\theta_{1}^{max}\right)p_{0}\;f\left(x\right)dx+\int_{\eta_{1}\left(Q\right)}^{Q}q_{1}\left(\theta_{0}^{+}\right)p_{0}\;f\left(x\right)dx. \end{array}$$ (43)

For Case 3, $1>\frac{\lambda }{\alpha p_{0}}>\frac{\lambda }{\left(\alpha +\beta \right)p_{0}}$,

$$\begin{array}{c} \begin{array}{c} h\left(Q\right)=\int_{0}^{\eta_{2}\left(Q\right)}q_{2}\left(\theta_{2}^{max}\right)p_{0}\;f\left(x\right)dx+\int_{\eta_{2}\left(Q\right)}^{\eta_{0}\left(Q\right)}q_{2}\left(\theta_{0}^{-}\right)p_{0}\;f\left(x\right)dx+\int_{\eta_{0}\left(Q\right)}^{Q}q_{1}\left(\theta_{0}^{+}\right)p_{0}\;f\left(x\right)dx, \end{array} \end{array}$$ (44)

where $\eta_{0}\left(Q\right)=\frac{Q}{1+\lambda }=\frac{Q}{\eta_{0}}$.

**Fig 1:**
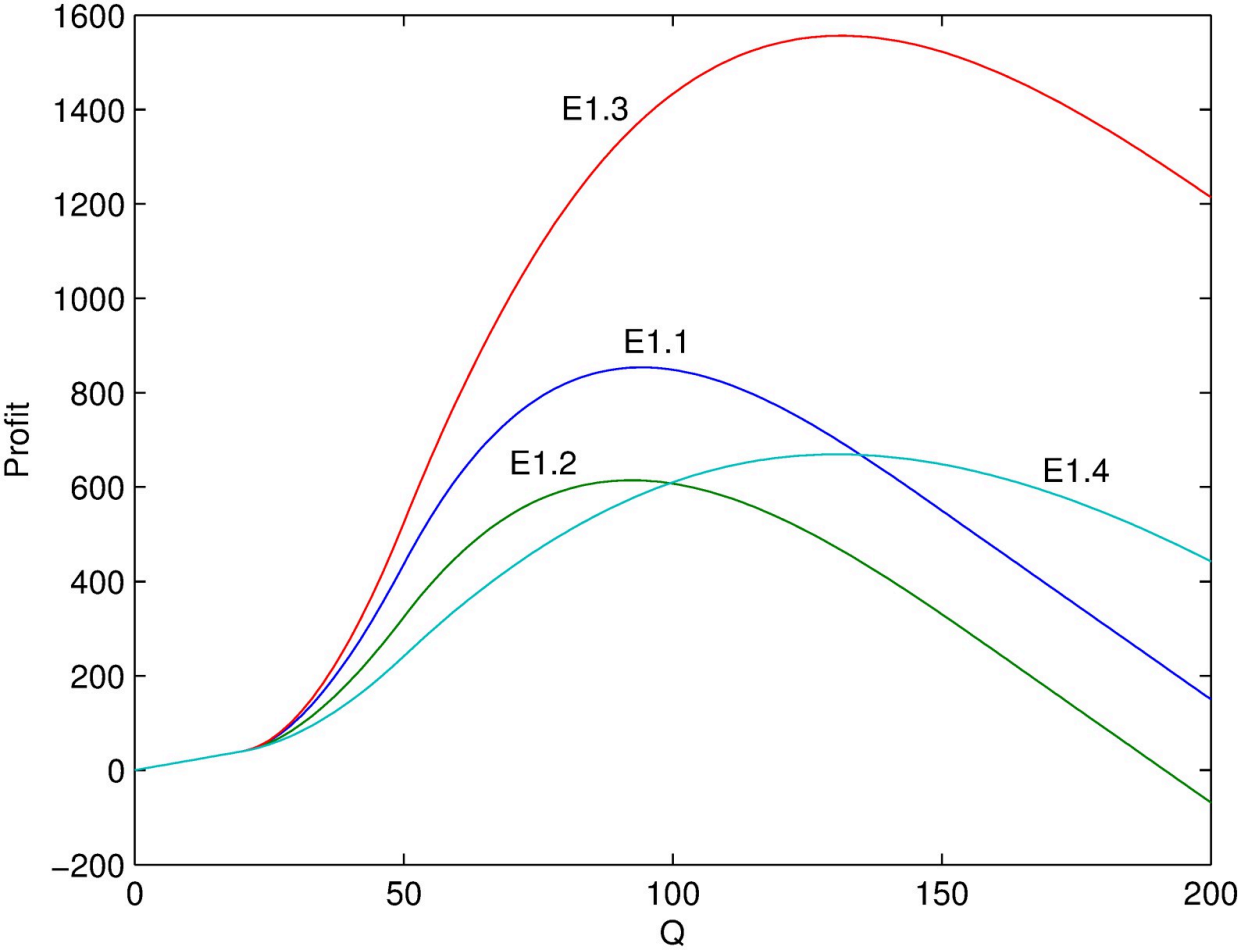
Comparison of maximal profits among examples E1.1-E1.4.

**Fig 2:**
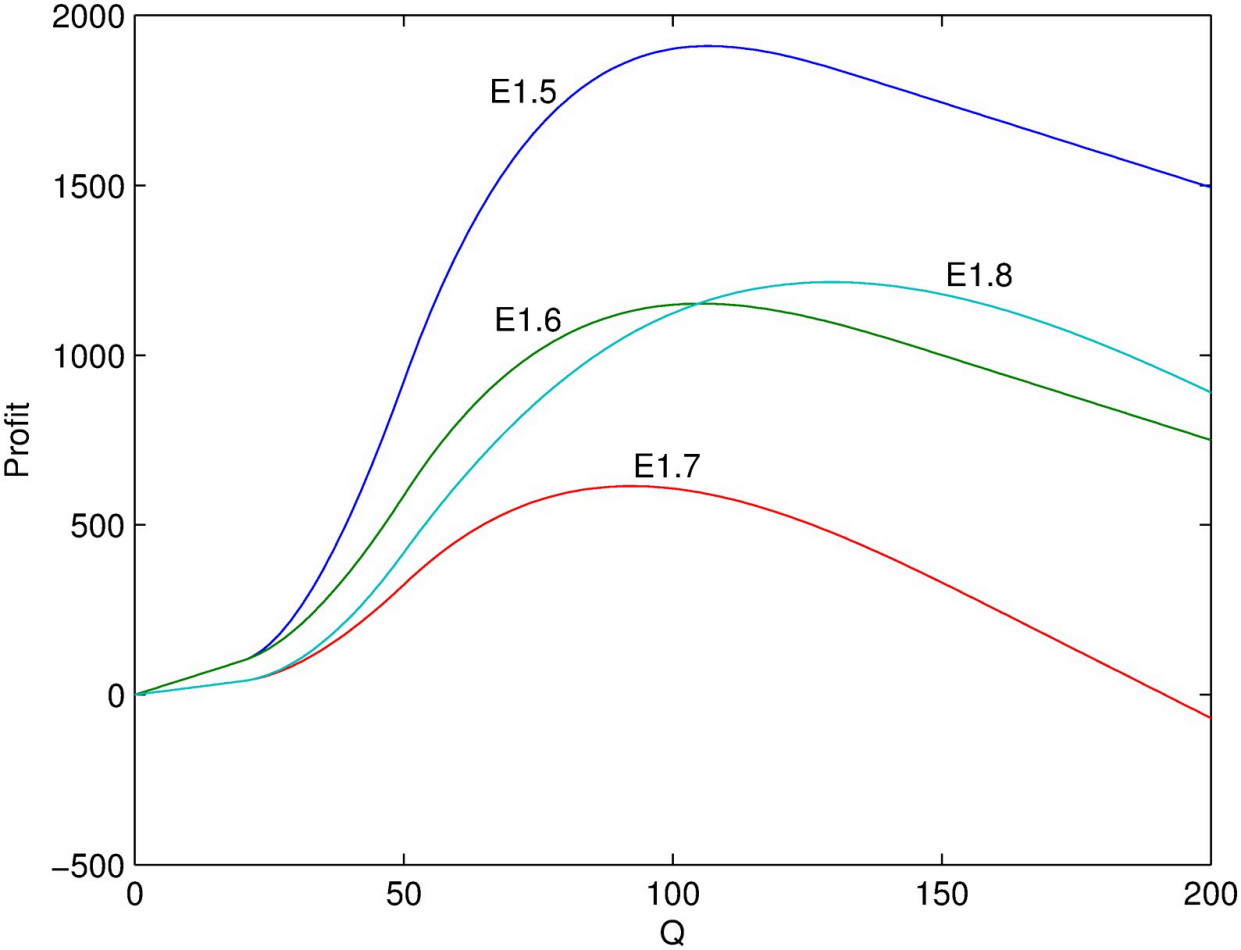
Comparison of maximal profits among examples E1.5-E1.8.

**Fig 3:**
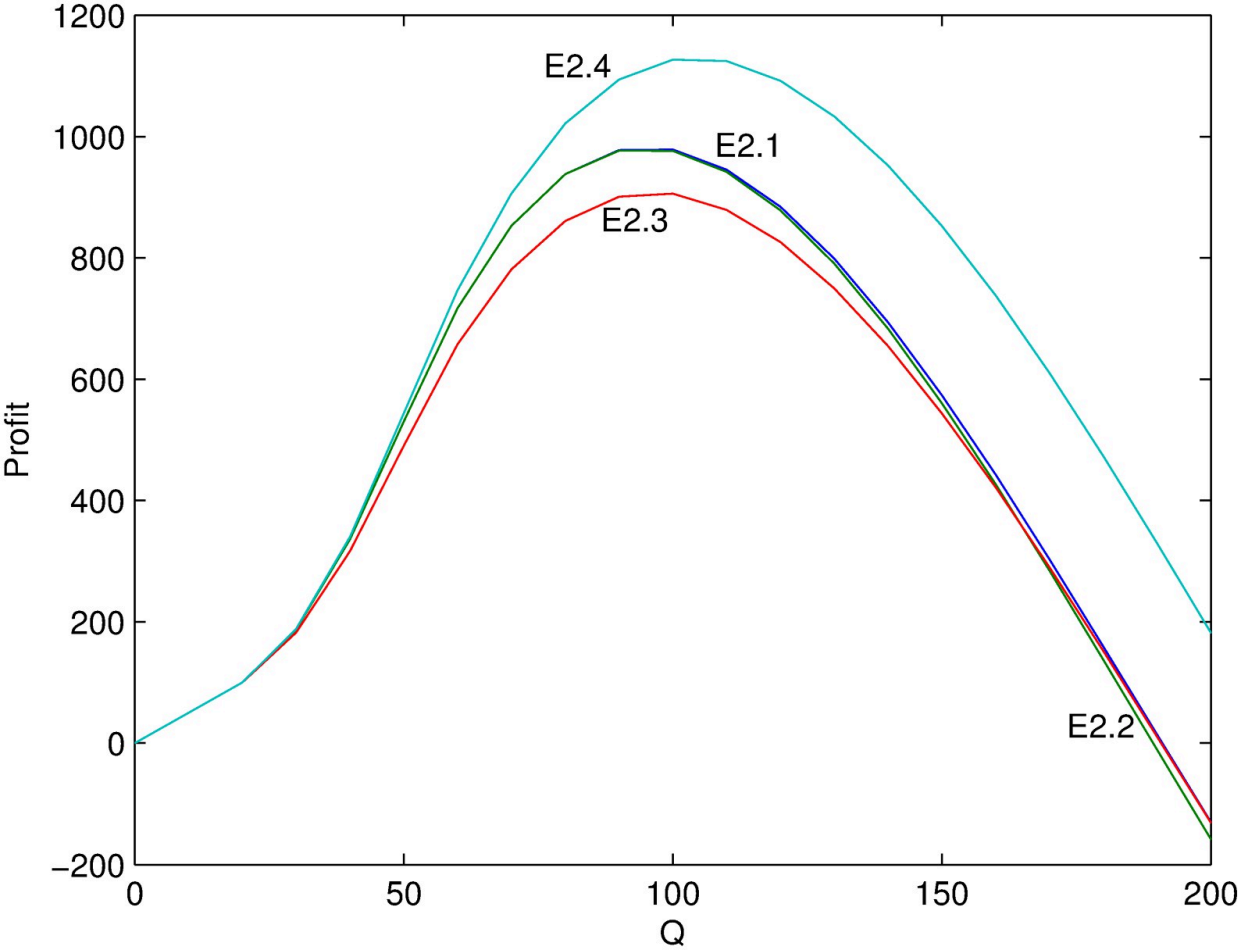
Comparison of maximal profits among examples E2.1-E2.4.

**Fig 4:**
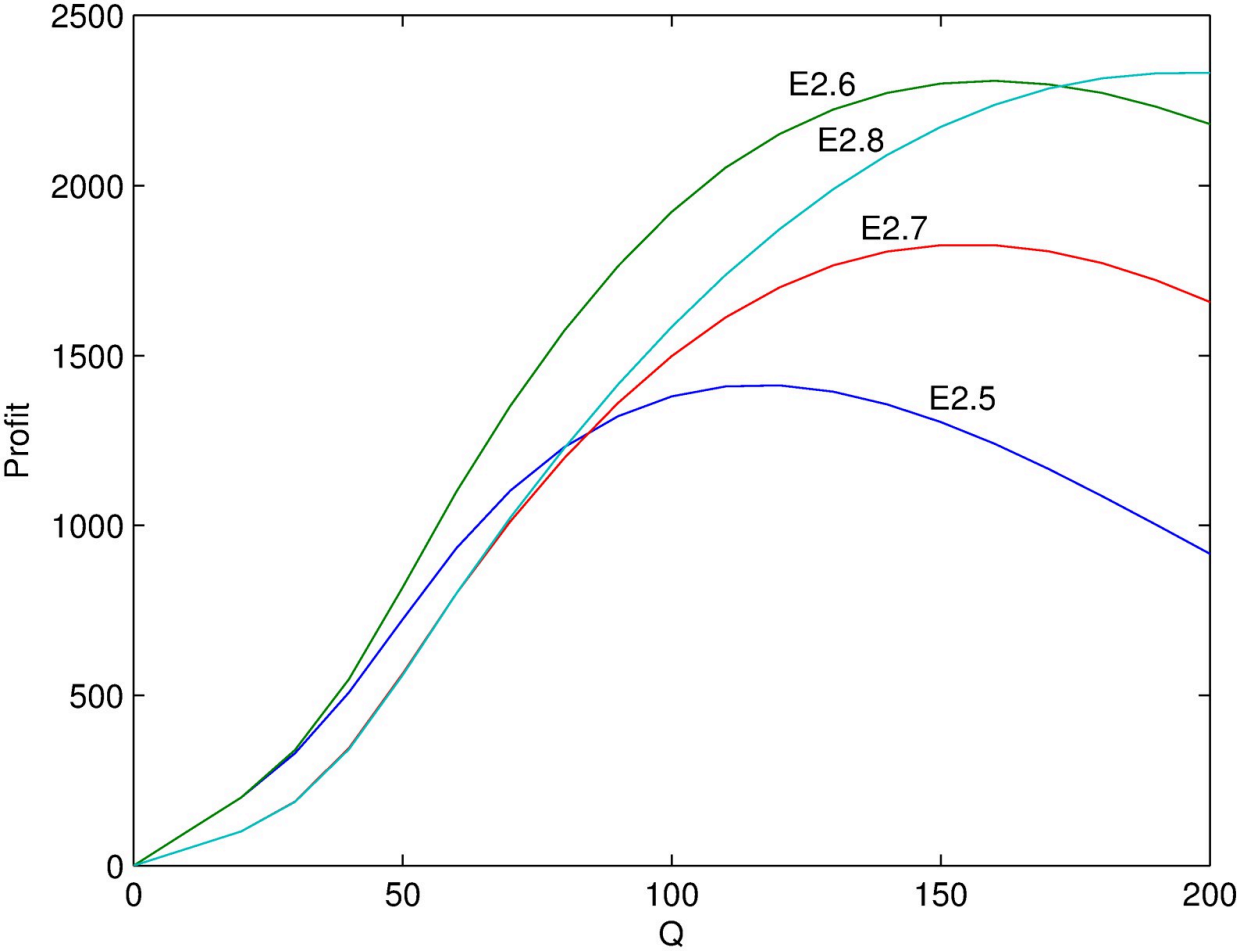
Comparison of maximal profits among examples E2.5-E2.8.

**Fig 5:**
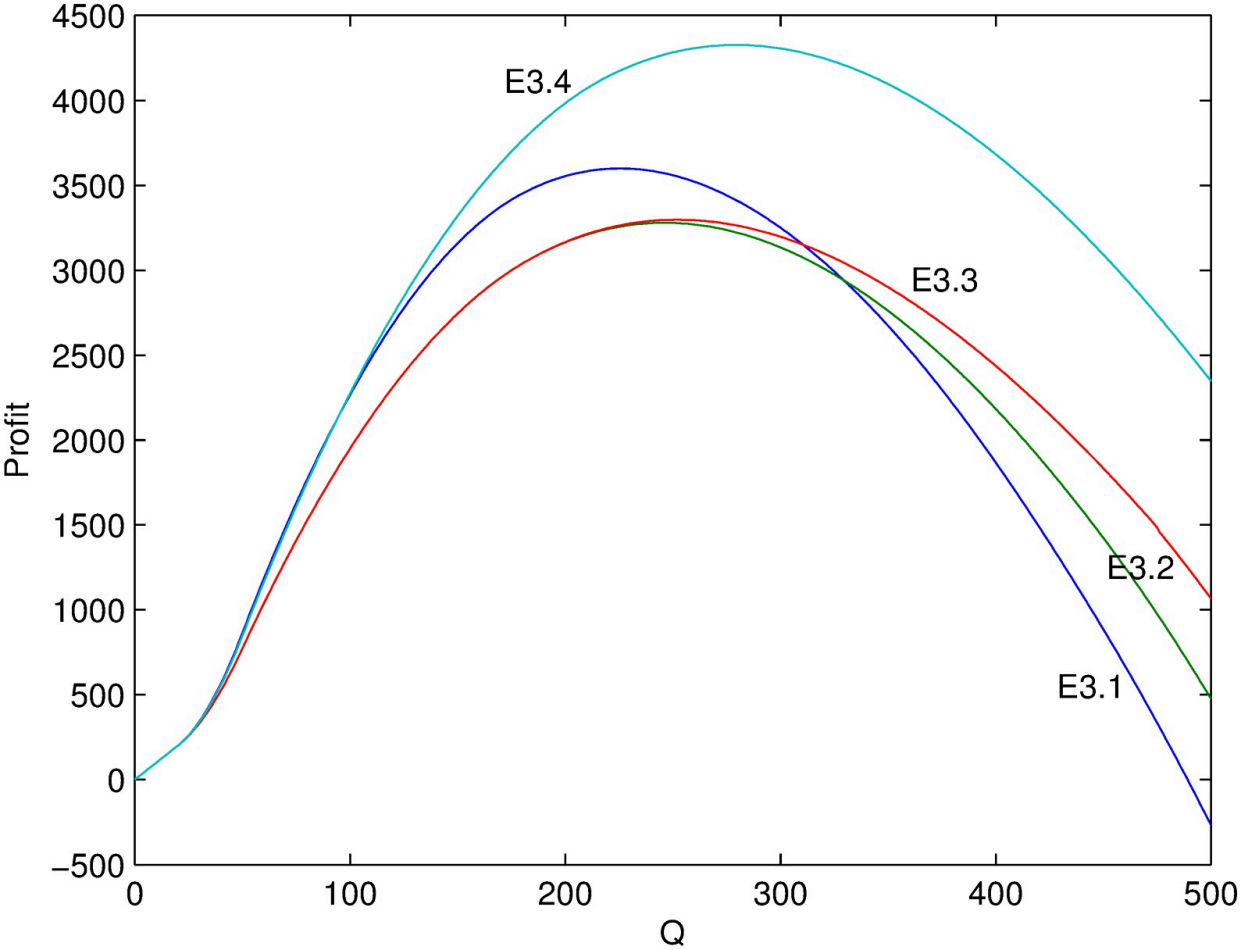
Comparison of maximal profits among examples E3.1-E3.4.

**Fig 6:**
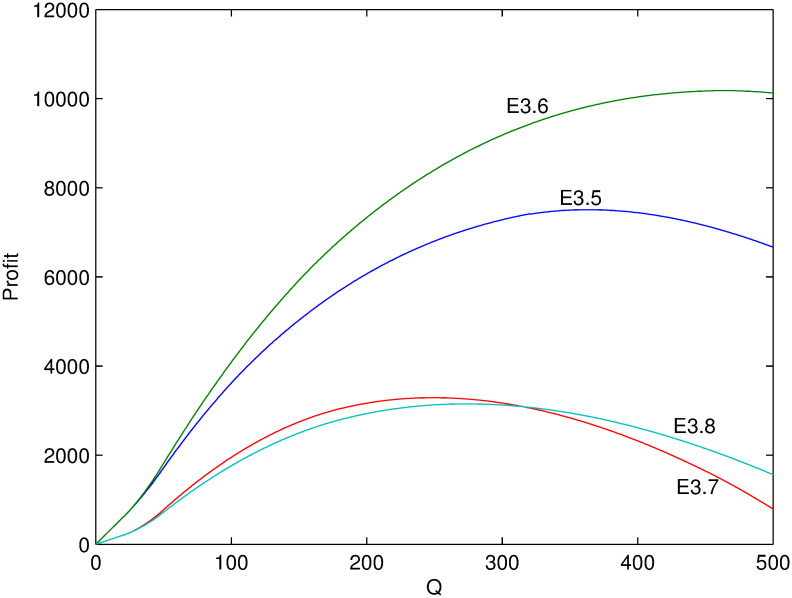
Comparison of maximal profits among examples E3.5-E3.8.

Next, several numerical examples (Tables 2–4) is present to test the proposed model. Figs 1–6 show these examples’ maximal profits as the initial quantity *Q* changes. For each example, one can order the optimal quantity *Q* to maximize the profit corresponding to its profit-quantity curve. For all the examples in this section, it is assumed that *x*_0_ is uniformly distributed on the interval [20, 50], that is, $f\left(x_{0}\right)=\frac{1}{30}$ and $F\left(x_{0}\right)=\frac{x_{0}-20}{30}$.

**Table 2:** Numerical examples for Case 1 where $\frac{\lambda }{\alpha p_{0}}>\frac{\lambda }{\left(\alpha +\beta \right)p_{0}}>1$.

No.	*α*	*β*	λ	*p*_0_	*c*	*η*_1_
E1.1	0.1	0.03	3	10	8	3
E1.2	0.15	0.03	3	10	8	3.25
E1.3	0.15	0.03	5	10	8	4.25
E1.4	0.45	0.03	5	10	8	5.57
E1.5	0.05	0.02	3	10	5	2.75
E1.6	0.1	0.05	3	10	5	3
E1.7	0.15	0.1	3	10	8	3.25
E1.8	0.2	0.12	5	10	8	4.5

**Table 3:** Numerical examples for Case 2 where $\frac{\lambda }{\alpha p_{0}}>1>\frac{\lambda }{\left(\alpha +\beta \right)p_{0}}$ and $\alpha \left(\alpha +\beta \right)p_{0}^{2}\geq \lambda^{2}$.

No.	*α*	*β*	λ	*p*_0_	*c*	*η*_1_	*η*_3_	*η*_2_
E2.1	0.12	0.1	3	20	15	3.7	4.13	4.7
E2.2	0.12	0.08	3	20	15	3.7	4.18	4.5
E2.3	0.14	0.08	3	20	15	3.9	4.01	4.7
E2.4	0.14	0.08	3.5	20	15	4.15	4.85	4.95
E2.5	0.15	0.1	3	20	10	4	4	5
E2.6	0.2	0.18	5	20	10	5.5	6.2	7.3
E2.7	0.25	0.2	6	20	15	6.5	7.16	8.5
E2.8	0.35	0.25	8	20	15	8.5	9.11	11

**Table 4:** Numerical examples for Case 3 where $1>\frac{\lambda }{\alpha p_{0}}>\frac{\lambda }{\left(\alpha +\beta \right)p_{0}}$.

No.	*α*	*β*	λ	*p*_0_	*c*	*η*_0_	*η*_2_
E3.1	0.3	0.25	8	50	40	9	17.5
E3.2	0.4	0.25	8	50	40	9	21.25
E3.3	0.4	0.35	8	50	40	9	23.75
E3.4	0.4	0.35	10	50	40	11	24.75
E3.5	0.3	0.2	5	50	20	6	16
E3.6	0.35	0.25	8	50	20	9	20
E3.7	0.4	0.3	8	50	40	9	22.5
E3.8	0.5	0.35	8	50	40	9	26.25

For examples E1.1-E1.8 where $\frac{\lambda }{\alpha p_{0}}>\frac{\lambda }{\left(\alpha +\beta \right)p_{0}}>1$, it isn’t optimal to decrease the in-season price based on previous analysis. The optimal in-season price should be larger than *p*_0_, that is, the adjusting parameter *θ* > 0. Thus, for any example, the maximal profit depends on *α* and λ but *β*. It is initial that the optimal profit is decreasing with *α* from examples E1.1 and E1.2 (or from examples E1.3 and E1.4). Because the larger value of *α*, the more price-sensitive the demand which means more demand lose with price increasing. Considering λ, the optimal profit is increasing with λ given any *Q* from examples E1.2 and E1.3. The reason is that there are enough potential demand when λ is larger and the retailer can adjust price as early as possible which is of course better than late adjustment with smaller λ.

Figs 1 and 2 show the corresponding maximal profits as the initial quantity *Q* changes. Comparing E1.4 with E1.2 (or E1.1), when *Q* is not large enough, e.g. *Q* < 100, the effect of *α* on profit is more obvious than that of λ and thus the profit of E1.2 (and E1.1) is larger than that of E1.4 given *Q*. When *Q* is large enough, e.g. *Q* > 150, the effect of *α* on profit is less obvious than that of λ and thus the profit of E1.2 (and E1.1) is smaller than that of E1.4. The same relation can be found from examples E1.8 and E1.6. However, the difference about effect on profit between *α* and λ is not obvious. For example, the profit of E1.5 is always larger than that of E1.8 which mainly results from the difference of *α*, although the values of λ are also different. While the profit of E1.7 is always no larger than that of E1.8 which mainly results from the difference of λ, although the values of *α* are also different. Of course, the unit purchase cost *c* also affects the result which is trivial.

For examples E2.1-E2.8, it satisfies the first condition of Case 2 where $\frac{\lambda }{\alpha p_{0}}>1>\frac{\lambda }{\left(\alpha +\beta \right)p_{0}}$ and $\alpha \left(\alpha +\beta \right)p_{0}^{2}\geq \lambda^{2}$. Figs 3 and 4 show their maximal profits as the initial quantity *Q* changes. First, the optimal profit is increasing with *β* from examples E2.1 and E2.2. Because the larger value of *β*, the more price-sensitive the demand which means that more new demand come with price decreasing. Second, from examples E2.2 and E2.3, the result shows that the profit is first decreasing and then increasing with *α*. Because it is optimal to increase price when *Q* is small and less demand lose with price increasing if *α* is small. Otherwise, if *α* is large, more demand lose with price increasing. When *Q* is large enough it is optimal to decrease price and the contrary is the case. Additionally, Fig 4 shows the similar relation with other figures including Figs 5 and 6 stated in the following.

For examples E3.1-E3.8, it satisfies the condition of Case 3 where $1>\frac{\lambda }{\alpha p_{0}}>\frac{\lambda }{\left(\alpha +\beta \right)p_{0}}$. Figs 5 and 6 show their maximal profits as the initial quantity *Q* changes. First, it is initial that the optimal profit is increasing with *β* from examples E3.2 and E3.3. Because the larger value of *β*, the more price-sensitive the demand which means that more new demand come with price decreasing. Second, in Case 3, price increasing or decreasing, i.e. *θ* > 0 or *θ* < 0, are both possibly optimal which mainly depends on the value of *Q*. Generally, when *Q* is small, it is optimal to increase the in-season price and thus *α* rather than *β* affects the profit. Under such situation, profit is decreasing with *α* (comparing E3.1 and E3.2). However, when *Q* is large enough, it is optimal to decrease the in-season price and thus both *α* and *β* affect the profit. Under such situation, profit is increasing with both *α* and *β* (comparing E3.1 with E3.2, or E3.2 with E3.3, or E3.1 with 3.3). The same relation can be found by comparing example E3.7 with example E3.8, which reflects the impact of price sensitivity on strategy selection. Additionally, the effects of *α* and λ on profit sometimes can offset each other. For example, the profits of E3.1 and E3.4 are almost the same when *Q* < 100 from Fig 5.

## Conclusion

In this paper, a joint inventory and price adjustment problem is formulated, which considers the extra demand caused by lower price. The model is developed mathematically and an analytical solution method is also proposed. In the model, it is first assumed that the inventory quantity is known and then the revenue is expressed as a function of price. The results show that the optimal in-season price mainly depends on the following three aspects, (1) the value of λ which reflects the proportion of the remaining demand when the price is equal to the initial price, (2) the value of *α* which reflects the price sensitivity, and (3) the value of *β* which reflects the effect of sales promotion. Briefly, the Theorems 1 and 2 state the effect of these values on the optimal price. An important managerial implication is that the retailer should gather the demand information about the price and raise the in-season price as soon as possible to gain more revenue when the price elasticity is small enough. Otherwise, when the price elasticity is larger, the retailer should maintain or decrease the price to gain more revenue.

In real world case, the information of demand is usually analyzed based on history data. However, this kind of information always ignores the extra demand. Consider the effect of extra demand, many scholars and practitioners research the “reservation policy” in practice [5]. Additionally, the proposed model is with practical significance even though without the consideration of extra demand (caused by promotion effect). Because the so-called demand function of price is fit through the historical data and it is usually described as smooth curve rather than piecewise in the literature. However, the real relationship between the demand and the price is surely more complicated and should usually be described as the joint of more than one smooth curve.

In this paper, the demand function of price is segmented which contributes to the solution for practical problems with more complex demand function. Therefore, it is a valuable direction to research more practical problem based on subdivided price interval and complicated demand function. Another direction for future research is a model where at time *t*_*r*_, one knows the probability distribution of the demand after *t*_*r*_ rather than the actual demand. The demand after *t*_*r*_ could have a distribution that depends on *t*_*r*_, the new selling price *p*_1_, and the demand prior to time *t*_*r*_. Given such a model, *t*_*r*_ could be a decision variable. One can also study the model with multiple reviews.

## Supporting information

S1 Appendix (ZIP)
