# Supplementary material for: Optimal decisions on price and inventory for a newsboy-type retailer with identifiable information and discount promotion
Source: PLoS One. 2023 Jul 21;18(7):e0288874. doi: 10.1371/journal.pone.0288874 (PMC10361536; doi:10.1371/journal.pone.0288874)
Supplement: S1 Appendix — (ZIP) [file pone.0288874.s001.zip › plos_S1.pdf]

Appendix A

In this appendix, Case 2a in Section 3 is explained.

Since it is the case that $\frac{\lambda}{\alpha p_0} > 1 > \frac{\lambda}{(\alpha+\beta)p_0}$ and $D(0) \le Q$, the retailer could increase or decrease the selling price to gain maximal revenue based on Theorem 1(c).

If the retailer increases the price, by Theorem 1(c), the retailer will choose $\theta = \theta_1^{\max}$ to gain maximal revenue equal to

$$R(\theta_1^{\max}) = (1+\lambda)x_0p_0 + \frac{(\lambda - \alpha p_0)^2 x_0}{4\alpha}. \tag{A1}$$

If the retailer decreases the price, by Theorem 1(c), the retailer will choose θ as follows.

$$\theta = \begin{cases} \theta_0^-, & D(0) \le Q \le \Delta_2 \\ \theta_2^{\max}, & \Delta_2 \le Q \end{cases}, \tag{A2}$$

where

$$\Delta_2 = (1+\lambda)x_0 + \frac{x_0}{2}[(\alpha+\beta)p_0 - \lambda]. \tag{A3}$$

Firstly, since $1 > \frac{\lambda}{(\alpha+\beta)p_0}$, $\Delta_2 > D(0) = (1+\lambda)x_0$. Secondly, when $Q = \Delta_2$, one can calculate that $\theta_0^- = \frac{1}{2}(\frac{\lambda}{(\alpha+\beta)p_0} - 1) = \theta_2^{\max}$ from Eqs 14 and 17. Using Lemma 2 and Theorem 1(c), one can get (A2).

If the retailer choose to decrease the price and it is the case that $D(0) \le Q \le \Delta_2$, the retailer will choose $\theta = \theta_0^- = \frac{(1+\lambda)x_0 - Q}{(\alpha+\beta)x_0p_0}$ from (A2) and Eq 17 to gain maximal revenue equal to

$$R(\theta_0^-) = q_2(\theta_0^-)p_0 = -\frac{Q^2}{(\alpha+\beta)x_0} + (p_0 + \frac{\lambda+2}{\alpha+\beta})Q - \frac{\lambda+1}{\alpha+\beta}x_0. \tag{A4}$$

If it is the case $\Delta_2 \le Q$, the retailer will choose $\theta = \theta_2^{\max} = \frac{1}{2}(\frac{\lambda}{(\alpha+\beta)p_0} - 1)$ from (A2) and Eq 14 to gain maximal revenue equal to

$$R(\theta_2^{\max}) = (1+\lambda)x_0p_0 + \frac{[\lambda - (\alpha+\beta)p_0]^2 x_0}{4(\alpha+\beta)}. \tag{A5}$$

When $\alpha(\alpha+\beta)p_0^2 \ge \lambda^2$, let $R(\theta_1^{\max}) \le R(\theta_0^-)$, one can get

$$\Delta_3 \le Q \le \Delta_4, \tag{A6}$$

where

$$\Delta_3 = \frac{(\alpha+\beta)x_0}{2}[p_0 + \frac{\lambda+2}{\alpha+\beta} - \sqrt{\frac{\beta}{\alpha+\beta}(p_0^2 - \frac{\lambda^2}{\alpha(\alpha+\beta)})}], \tag{A7}$$

$$\Delta_4 = \frac{(\alpha+\beta)x_0}{2}[p_0 + \frac{\lambda+2}{\alpha+\beta} + \sqrt{\frac{\beta}{\alpha+\beta}(p_0^2 - \frac{\lambda^2}{\alpha(\alpha+\beta)})}]. \tag{A8}$$

Because when $Q = D(0)$, it is the case that

$$R(\theta_0) = (1+\lambda)x_0p_0 < (1+\lambda)x_0p_0 + \frac{(\lambda - \alpha p_0)^2 x_0}{4\alpha} = R(\theta_1^{\max}), \tag{A9}$$

and when $Q = \Delta_2$, it is the case that

$$R(\theta_0^-) = R(\theta_2^{\max}) \geq R(\theta_1^{\max}). \tag{A10}$$

Moreover, $R(\theta_0^-)$ is monotone increasing in $Q \in [D(0), \frac{(\alpha+\beta)p_0-\lambda}{2}x_0]$, and monotone decreasing in $Q \in [\frac{(\alpha+\beta)p_0-\lambda}{2}x_0, +\infty]$. Thus, based on $\Delta_2 > \frac{(\alpha+\beta)p_0-\lambda}{2}x_0$, one can show that

$$\Delta_4 \geq \Delta_2 \geq \Delta_3 \geq D(0). \tag{A11}$$

At the same time, when $\alpha(\alpha+\beta)p_0^2 \geq \lambda^2$, one can show that

$$R(\theta_2^{\max}) \geq R(\theta_1^{\max}). \tag{A12}$$

That is, when $\alpha(\alpha+\beta)p_0^2 \geq \lambda^2$, the retailer should choose $\theta^*(x_0, Q)$ as follows in Case 2a.

$$\theta^*(x_0, Q) = \begin{cases} \theta_1^{\max}, & D(0) \leq Q \leq \Delta_3 \\ \theta_0^-, & \Delta_3 \leq Q \leq \Delta_2 \\ \theta_2^{\max}, & \Delta_2 \leq Q \end{cases}, \tag{A13}$$

and the corresponding revenue will be

$$g(x_0, Q) = \begin{cases} q_1(\theta_1^{\max})p_0, & D(0) \leq Q \leq \Delta_3 \\ q_2(\theta_0^-)p_0, & \Delta_3 \leq Q \leq \Delta_2 \\ q_2(\theta_2^{\max})p_0, & \Delta_2 \leq Q \end{cases}. \tag{A14}$$

When $\alpha(\alpha+\beta)p_0^2 < \lambda^2$, let $R(\theta_1^{\max}) \leq R(\theta_0^-)$, there will be no solution to this inequality. In other words, when $\alpha(\alpha+\beta)p_0^2 < \lambda^2$ it is always the case that $R(\theta_1^{\max}) > R(\theta_0^-)$.

At the same time, when $\alpha(\alpha+\beta)p_0^2 < \lambda^2$, one can show that

$$R(\theta_2^{\max}) < R(\theta_1^{\max}). \tag{A15}$$

Thus, when $\alpha(\alpha+\beta)p_0^2 < \lambda^2$, the retailer should choose $\theta^*(x_0, Q)$ as follows in Case 2a.

$$\theta^*(x_0, Q) = \theta_1^{\max}, \tag{A16}$$

and the corresponding revenue will be

$$g(x_0, Q) = q_1(\theta_1^{\max})p_0. \tag{A17}$$
